# A biomonitoring study on blood levels of beta-hexachlorocyclohexane among people living close to an industrial area

**DOI:** 10.1186/1476-069X-12-57

**Published:** 2013-07-16

**Authors:** Daniela Porta, Fiorella Fantini, Elena De Felip, Francesco Blasetti, Annalisa Abballe, Valerio Dell’Orco, Valeria Fano, Anna Maria Ingelido, Silvia Narduzzi, Francesco Forastiere

**Affiliations:** 1 Department of Epidemiology, Lazio Regional Health Service- ASL RME, Rome, Italy; 2 Department of Prevention, Local Health Unit Roma G, Colleferro, Italy; 3 Department of the Environment and Primary Prevention, National Institute for Health, Rome, Italy; 4 Local Health Unit Rome D, Rome, Italy

**Keywords:** Human biomonitoring, Beta-HCH, Persistent organic pollutants, Water contamination, Food contamination

## Abstract

**Background:**

A chemical plant manufacturing pesticides has been operating since the 1950’s in the Sacco River Valley (Central Italy). In 2005, high beta-hexachlorocyclohexane (Beta-HCH) concentrations were found in milk of cows raised and fed near the river. We report the results of a biomonitoring study conducted in this region to evaluate the body burden of Beta-HCH and to identify the determinants of the human contamination.

**Methods:**

We defined four residential areas by their distance from the chemical plant and the river, and selected a stratified random sample of 626 people aged 25–64 years. We evaluated the association, in terms of the geometric mean ratio (GMR), between several potential determinants and Beta-HCH serum concentrations using multivariate linear regression analysis.

**Results:**

Two hundred forty-six serum samples were analysed to assess Beta-HCH levels (mean concentration: 99 ng/g lipid; Standard Deviation: 121; Geometric Mean: 60.6; Geometric Standard Deviation: 2.65). We found a strong association between Beta-HCH and living in the area close to the river (GMR: 2.00; 95%CI: 1.36-2.94). Beta-HCH levels were also associated with age, level of education, use of private wells and consumption of local food.

**Conclusions:**

The results suggest that people living close to the river may have been contaminated by Beta-HCH, most likely through water from private wells and privately grown food. A programme of epidemiological and clinical surveillance is on-going on this population.

## Background

β-hexachlorocyclohexane (β-HCH) is an isomer of hexachlorocyclohexane (HCH) [1]. It is a persistent organochloride, a byproduct of the production of lindane (γ-HCH), an insecticide widely used during the 1960’s and 1970’s. The production of lindane, and then of β-HCH, has been prohibited since the beginning of this century in several countries, including Italy (Regulation (EC) No 850/2004), and has been included in the list of the 9 new POPs (persistent organic pollutants) at the Stockholm Convention [2]. Compared to other isomers of HCH, β-HCH has stronger lipophilic properties and persists longer in the environment. It can be absorbed by humans through contamination of the food chain and it is bioaccumulated in body fat [1]. Moreover, it has been shown that β-HCH can pass through the placenta and accumulates in breast milk [1,3]. There are only few information regarding the half-life of β-HCH, but it is estimated between 1 and several years [4].

Like other persistent organic pollutants, β-HCH is suspected to be harmful to humans. However, knowledge of its effects on human health is limited and controversial and in many cases the evidence is based only on studies of workers employed in the production and use of lindane [5]. The most frequently reported effects are related to neurological disorders [6,7], endocrine disruption [8,9], reproductive disorders [10,11], cardiovascular effects [12] and cancer [13,14].

In 2005, during a random national survey on chemical contamination of raw cow’s milk, products from a farm located in a rural area along the Sacco river (Sacco Valley, Central Italy) were found to be contaminated with β-HCH [15]. A level of 0.062 mg/kg was found, which is more than 20 times legal levels, i.e. 0.003 mg/kg for milk, according to European law (Directives 97/41/CE, 1999/65/CE, 1999/71/CE). Subsequent analyses of milk from other farms and samples of the soil and the river water clearly indicated extensive environmental pollution in the area. β-HCH was found in water close to the industrial plant (from 0.34 to 7.00 μg/L) and in water and river sediment downstream of the plant (from 0.016 to 1.46 mg/Kg of sandy soil (ss)). It was also found in 5 out of 35 water wells in Colleferro (the municipality where the industrial plant and the landfills are located), and in the riparian areas up to 200 meters from the river (from 0.015 to 1.73 mg/Kg ss) (Technical report on the Monitoring of environmental matrix and health effects of the population residing in the Sacco Valley. DGR N.540, 19.05.2005 and N.550, 27.05.2005).

The Sacco river valley is characterized by the presence of a large industrial conglomerate, including a chemical plant located in the city of Colleferro, which has produced lindane since the 50’s. Contamination was discovered in two landfills within the industrial area in 1990 and reports from the population indicate that they had been present since the 1960’s, but no evidence of animal or human contamination was investigated at that time.

The sequence of the contamination we postulated was the following: continuous leakage of chemicals from the landfills, and from runoff waters of the soil surrounding the plant, contaminated the nearby river through a hidden drainage ditch. The river water was used to irrigate the crops and periodical winter river flooding increased the chemical contamination of the cultivated soil. Animals were contaminated because they were fed with fodder grown in the contaminated area.

We hypothesized subsequent contamination of the people residing in the area in two main ways due to the rural character of the area: the use of water from private wells, which were widespread in the area, and the consumption of food (such as meats, milk and dairy products) produced in the contaminated farms, by the producers themselves, but also by people who bought them directly from producers.

To confirm our hypothesis, we conducted a biomonitoring study to evaluate the body burden of blood concentrations of β-HCH in the population living in the Sacco Valley, and to identify the possible factors associated with the contamination. In this paper we report the results of the biomonitoring campaign.

## Methods

### Area under study

**Figure 1:**
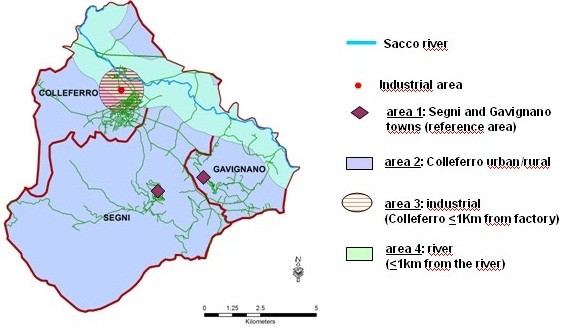
Description of the 4 areas under study.

We conducted a biomonitoring study between September 2006 and August 2007. The area under study is shown in Figure 1. It includes the territory of three small towns (Colleferro, Segni and Gavignano) in the province of Rome (central Italy) where the industrial area of Colleferro and the Sacco river are located. We divided this area into 4 sub-areas according to their supposed level of exposure, based on their distances from the chemical factory (that produced lindane) and from the river (found to be very contaminated). Area 1 included the developed area of Segni and Gavignano, located in the hills and considered the reference area, area 2 (Colleferro urban/rural) included the part of the town of Colleferro (further from the industrial area) and the surrounding rural areas of Colleferro, Segni and Gavignano, area 3 (industrial) included the 1 km radial buffer around the chemical factory; area 4 (river) included the 1 km linear buffer along the river where many farms were located. In case of overlap between sub-areas, we classified people in the one that anticipated the higher exposure level (from area 4 to area 1).

### Study population

The population under study was a random sample of the residents of each sub-area. We collected the records of the population living in the municipality of Colleferro, Segni and Gavignano from the population register and we selected a random sample stratified by gender and age class (25–44, 45–64) with STATA 8. Among the sampled people, the criteria of eligibility to give the blood sample were: to be resident of one of the four sub-areas for a minimum of 15 years, not having lost more than 10 kilograms in the last three years, and an absence of cancer. Women who breastfed in the past 15 years were excluded as well. We had the intention (and the resources) to analyze a maximum of 250 blood tests. Therefore, because of the strict eligibility criteria, we increased the sample size to be sure to reach that number. In particular, we over-sampled people from the area close to the river (area 4), which was expected to be the most contaminated, and women, which had more strict selection criteria.

Individual data were available only for authorized personnel and health data were recorded separately from demographic information.

The research protocol was approved by the ethics committee of the Local Health Unit RMG in Colleferro. All participants gave their consent.

### Data collection

Subjects were invited to participate by mail, in person by their general practitioner, or by phone. A questionnaire was administered to all the participants to collect information on personal and behavioural characteristics: sex, age, education level, sub-area of residence (as defined above), distance between address of residence and river, use of private well water for drinking, cooking, or bathing, and use of water from other private wells, origin of food, and smoking habits. Regarding origin of food, we did not collect information about the frequency with which the food from any of the sources considered had been consumed. We assigned the classification of the origin giving the priority to the own production’s origin, then the local, and finally the commercial one. All the variables mentioned above were selected as potential determinants of an individual’s blood level of pollutants [16,17].

A 40 ml blood sample was taken from all people who met eligibility criteria and who consented to participate, in order to measure the serum concentrations of β-HCH and other organic pollutants (these results are beyond the scope of this paper, but some descriptive data are presented in the Additional file 1).

### Laboratory analysis

The blood samples were stored at -20°C and β-HCH serum levels measured at the Department of the Environment and Primary Prevention, National Institute for Health, in Rome [18]. In brief, an aliquot of 10 mL of each serum sample was fortified with 13C-labelled β-HCH and allowed to rest overnight. Then samples were added with a mixture of formic acid and 2-propanol, sonicated, and extracted by manual shaking with n-hexane. The organic phase was removed after centrifugation, and the extraction process was performed twice. The organic fractions were mixed together into a centrifuge tube, concentrated sulphuric acid was added, then was shaken and separated by centrifugation. The extracts were reduced in volume, purified on activated neutral alumina, concentrated and transferred to 1-mL autosampler vials for quantification. Instrumental analysis was carried out by ion trap mass spectrometry, using a Thermo Scientific PolarisQ GC-ion trap in the MS/MS mode. Data were processed using XCALIBUR software. Recovery rates ranged from 75-110%. Analytical reliability was evaluated with a method validated in-house [18]. Lipid levels (cholesterol, phospholipid, and triglycerides) were determined using enzymatic methods [18].

### Statistical analysis

Individual characteristics of the 4 sub-areas studied, considered as potential determinants of the levels of β-HCH, were described. Serum concentrations of the pollutant were measured using geometric means (GM) and geometric standard deviation (GSD) for the whole population, and by the aforementioned potential determinants. To test the heterogeneity across GMs we performed univariate Anova models on the logarithmic transformation of β-HCH (log- β-HCH). The log-β-HCH was also used to perform multivariate linear regression models, so the associations were expressed as Geometric Mean Ratios (GMR) (i.e. the exponent of the linear regression coefficients) and 95% Confidence Intervals (95%CI).

We initially included in the multivariate linear regression model all potential determinants: gender (males, females), age group (20–34 years, 35–44, 45–54, 55–64), education level (primary school, high school, university degree), sub-area of residence (area 1, 2 3, 4), distance from the river (> 5 Km, 2–5 Km, 1–2 Km, 500 m-1 Km, < 500 m), any use of private well water (at least one among drinking, cooking, bathing, and use of someone else’s private well), provenience of food (commercial, local, own production), and smoking habits. Since the sub-area of residence and distance from the river were highly correlated, we considered in the model only the former. In the final model we also excluded smoking habit, as it was not associated with levels of β-HCH. Since the residents of area 4 showed the highest levels of β-HCH, we conducted a stratified multivariate regression analysis considering two groups: subjects residing in area 4 and those residing in the other 3 sub-areas (area 1–3). We also performed separate analyses including the origin of each food (eggs, milk, cheese, beef, chicken, liver, pork, raw and cooked vegetables). In the additional material we report the distribution of the sources of each food (Additional file 2), the data description of β-HCH by source of each food (Additional file 3) and the effect of the foods on the levels of β-HCH, estimated running a food at a time, instead of the variable provenience of food, in the final multivariate model, as described before (Additional file 4). We also ran a multivariate model with all the foods together, excluding the food with an association not statistically significant in the model with the single food (liver, raw vegetables and cooked vegetables), and once a time the food with a p-value >0.2 (eggs, milk, cheese, pork). The results of the model with the remaining foods (beef and chicken meat) is reported below.

All the analyses were performed with STATA 8.

## Results

We randomly selected and invited 626 persons to participate in the study, stratified by sub-area of residence, gender and age. There were 509 respondents to the questionnaire (81.3%), 404 (79.4 %) of whom were also eligible to provide a blood sample. These subjects were invited to give a blood sample until the predetermined number of samples was reached. In the end, 246 subjects (response rate: 60.9%) provided blood samples: 30 resided in area 1, 52 in area 2, 47 in area 3 and 117 in area 4 (Table 1).

**Table 1:** Population invited to the survey, respondents to questionnaire, eligible to and giving a blood sample, by sub-area of residence, gender and age

	**Males**				**Females**				**Total**	
	**25-44**		**45-64**		**25-44**		**45-64**			
	**N**	**%**	**N**	**%**	**N**	**%**	**N**	**%**	**N**	**%**
**Area 1**										
Subjects invited to the survey	13	100	13	100	20	100	20	100	66	100
Subjects responding of the questionnaire*	11	84.6	9	69.2	7	35.0	16	80.0	43	65.2
Subjects eligible to give blood sample	10	100	10	100	5	100	14	100	39	100
Subjects providing a valid blood sample**	8	80.0	9	90.0	5	100	8	57.1	30	76.9
**Area 2**										
Subjects invited to the survey	25	100	25	100	40	100	40	100	130	100
Subjects responding of the questionnaire*	22	88.0	25	100	31	77.5	39	97.5	117	90.0
Subjects eligible to give blood sample	15	100	27	100	16	100	34	100	92	100
Subjects providing a valid blood sample**	12	80.0	13	48.1	12	75.0	15	44.1	52	56.5
**Area 3**										
Subjects invited to the survey	25	100	25	100	40	100	40	100	130	100
Subjects responding of the questionnaire*	13	52.0	18	72.0	28	70.0	32	80.0	91	70.0
Subjects eligible to give blood sample	11	100	18	100	12	100	28	100	69	100
Subjects providing a valid blood sample**	11	100	13	72.2	9	75.0	14	50.0	47	68.1
**Area 4**										
Subjects invited to the survey	60	100	60	100	90	100	90	100	300	100
Subjects responding of the questionnaire*	56	93.3	60	100	78	87	64	71.1	258	86
Subjects eligible to give blood sample	50	100	58	100	37	100	59	100	204	100
Subjects providing a valid blood sample**	27	54	34	58.6	29	78	27	45.8	117	57.4
**Total**										
Subjects invited to the survey	123	100	123	100	190	100	190	100	626	100
Subjects responding of the questionnaire*	102	82.9	112	91.1	144	76	151	79.5	509	81.3
Subjects eligible to give blood sample	86	100	113	100	70	100	135	100	404	100
Subjects providing a valid blood sample**	58	67.4	69	61.1	55	79	64	47.4	246	60.9

* Percentage of the subjects invited to the survey.

** Percentage of the subjects eligible to give blood sample.

Table 2 shows the characteristics of the population that provided blood samples, by sub-area of residence. Residents of area 4 were more likely to use private well water and to eat food grown locally or of their own production than were the people living in the other sub-areas. People living close to the river (area 4) were less educated than people living in the other sub-areas, especially compared to those in the industrial and reference areas. The subjects that provided blood samples did not differ from those who did not provide blood for all the characteristics shown in Table 2, with the only exception of gender as there were fewer females due to the selection criteria related to breastfeeding.

**Table 2:** Characteristics of the population who provided a blood sample by area of residence

**Variables**	**Area 1**	**Area 2**	**Area 3**	**Area 4**	**Total**
	**N (%)**	**N (%)**	**N (%)**	**N (%)**	**N (%)**
**TOTAL**	**30 (100)**	**52 (100)**	**47 (100)**	**117 (100)**	**246 (100)**
**Sex**					
Males	17 (56.7)	25 (48.1)	24 (51.1)	61 (52.1)	127 (51.6)
Females	13 (43.3)	27 (51.9)	23 (48.9)	56 (47.9)	119 (48.4)
**Age group (years)**					
25-34	4 (13.3)	12 (23.1)	6 (12.8)	27 (23.1)	49 (19.9)
35-44	9 (30.0)	12 (23.1)	14 (29.8)	29 (24.8)	64 (26.0)
45-54	6 (20.0)	13 (25.0)	9 (19.1)	27 (23.1)	55 (22.4)
55-64	11 (36.7)	15 (28.8)	18 (38.3)	34 (29.1)	78 (31.7)
**Level of education**					
Primary school	9 (30.0)	19 (36.5)	20 (42.5)	75 (61.5)	120 (48.8)
High school	18 (60.0)	30 (57.7)	21 (44.7)	39 (33.3)	108 (43.9)
University degree	3 (10.0)	3 (5.77)	6 (12.8)	6 (5.13)	18 (7.32)
**Smoking**					
Never	14 (46.7)	23 (44.2)	19 (40.4)	78 (66.7)	134 (54.5)
Yes	16 (53.3)	29 (55.8)	28 (59.6)	39 (33.3)	112 (45.5)
**Distance from the river**					
> 5 km	22 (73.3)	6 (11.5)	0 (0.0)	4 (3.4)	32 (13.0)
> 2–5 km	5 (16.7)	22 (42.3)	4 (8.5)	4 (3.4)	35 (14.2)
1-2 km	1 (3.3)	16 (30.8)	17 (36.2)	42 (35.9)	76 (30.9)
500 m-1 km	0 (0.0)	5 (9.6)	19 (40.4)	37 (31.6)	61 (24.8)
< 500 m	2 (6.7)	3 (5.8)	7 (14.9)	30 (25.6)	42 (17.1)
Use of well water*					
No	29 (96.7)	46 (88.5)	45 (95.7)	31 (26.5)	151 (61.4)
For drinking	1 (3.3)	1 (1.9)	0 (0.0)	28 (23.9)	30 (12.2)
For cooking	0 (0.0)	6 (11.5)	2 (4.3)	69 (59.0)	77 (31.3)
For personal cleaning	0 (0.0)	5 (9.6)	0 (0.0)	81 (69.2)	86 (35.0)
Someone else’s well	1 (3.3)	0 (0.0)	0 (0.0)	3 (2.6)	4 (1.6)
Any use	1 (3.3)	6 (11.5)	2 (4.3)	86 (73.5)	95 (38.6)
**Provenience of food**					
Commercial	11 (36.7)	16 (30.8)	24 (51.1)	14 (12.0)	65 (26.4)
Local	10 (33.3)	25 (48.1)	19 (40.4)	55 (47.0)	109 (44.3)
Own production	9 (30.0)	11 (21.2)	4 (8.5)	48 (41.0)	72 (29.3)

Area 1: reference; Area 2: Colleferro urban/rural; Area 3: industrial; Area 4: river.

* Responses are not mutually exclusive.

Tables 3 reports the geometric mean (GM) (and GSD) of ß-HCH serum levels by sub-area of residence, together with the p-value from the Fisher test. The GM in the total population was 60.6 ng/g lipid. The GM of ß-HCH increased with age (38.8 ng/g lipid in 20–34 year olds, 99.3 ng/g lipid in 55–64 year olds), and showed striking differences by sub-area of residence, with subjects from area 4 having much higher levels (97.7 ng/g lipid) than those from the other sub-areas (34.5 ng/g lipid (area 1), 36.1 ng/g lipid (area 2) and 43.8 ng/g lipid (area 3)). This result was confirmed by the increase of ß-HCH serum concentrations with distance from the river (GM= 40.4 ng/g lipid for distances over 5 km versus 77.6 ng/g lipid for distances under 500 m). The ß-HCH serum levels were lower with increasing levels of education (GMs ranged from 89.9 ng/g lipid in people who attended up to primary school, to 38.3 ng/g lipid in people who graduated). Higher levels of the pollutant were found in subjects using private well water (GM for any use= 101.1 ng/g lipid vs no use 42.9 ng/g lipid). Finally, the concentration of ß-HCH was more than doubled in subjects who consumed local or food of their own production (GM=62.4 and 81.9 ng/g lipid, respectively) compared to subjects who consumed only commercial food (41.4 ng/g lipid).

**Table 3:** Descriptive data (GM and GSD) of ß -HCH (ng/g lipid) by the main population characteristics

	**N**	**GM**	**±**	**GSD**	pvalue*
**TOTAL**	216	60.58	±	2.65	
**Sex**					0.2209
Males	110	55.93	±	2.68	
Females	106	65.82	±	2.62	
**Age group (years)**					<0.001
25-34	42	38.75	±	3.04	
35-44	57	41.94	±	2.48	
45-54	49	68.69	±	2.31	
55-64	68	99.25	±	2.18	
**Level of education**					<0.001
Primary school	108	89.86	±	2.56	
High school	91	41.33	±	2.38	
University degree	17	38.31	±	2.05	
**Smoking**					0.2003
Never	124	65.19	±	2.78	
Yes	92	54.88	±	2.47	
**Area of residence**					<0.001
Area 1 (reference)	25	34.47	±	1.91	
Area 2 (Colleferro urban/rural)	46	36.05	±	2.17	
Area 3 (industrial)	39	43.82	±	2.20	
Area 4 (river)	106	97.65	±	2.59	
**Distance from the river**					<0.001
> 5 km	28	40.36	±	1.80	
> 2–5 km	30	37.92	±	2.65	
1-2 km	66	61.01	±	2.32	
500 m-1 km	55	80.73	±	2.82	
< 500 m	37	77.61	±	3.07	
**Use of private well water for drinking**					<0.001
No	189	55.10	±	2.54	
Yes	27	117.7	±	2.81	
**Use of private well water for cooking**					<0.001
No	146	46.39	±	2.48	
Yes	70	105.7	±	2.39	
**Use of private well water for personal cleaning**					<0.001
No	138	44.69	±	2.35	
Yes	78	103.8	±	2.58	
**Use of someone else’s well water**					0.0666
No	212	59.58	±	2.61	
Yes	4	146.9	±	4.45	
**Any use of well water**					<0.001
No	129	42.87	±	2.32	
Yes	87	101.1	±	2.55	
**Provenience of food**					0.0006
Commercial	56	41.43	±	2.05	
Local	99	62.38	±	2.74	
Own production	61	81.87	±	2.79	

GM: Geometric Mean; GSD: Geometric Standard Deviation.

* p-value from F-test performed on the log distribution of ß-HC.

Table 4 shows the results of the multivariate linear regression model. The ß-HCH level raised with age and was particularly high in the oldest age group compared to 20–34 year old (GMR: 2.76; 95%CI: 2.04-3.74). It was significantly higher in area 4 than in area 1 (GMR: 2.00; 95%CI: 1.36-2.94), and in subjects who used private well water for any reason (GMR: 1.42; 95%CI: 1.08-1.88). Subjects with higher education were less likely to have high ß-HCH levels than less educated ones. The geometric mean ratio was higher for subjects who consumed local food or food they produced than in subjects who consumed commercial food, but the association did not reach statistical significance. The analysis stratified by sub-area confirmed the same results of the overall analysis in area 4, with a stronger effect of privately produced food (GMR: 2.27; 95%CI: 1.24-4.13). In the other sub-areas combined (areas 1, 2 and 3), increasing age and female gender were the main determinants of high level of ß-HCH. The model with the consume of beef and chicken, instead of provenience of food, showed an association between levels of ß-HCH and privately grown beef and chicken (GMR_beef_: 1.88; 95%CI: 1.35-2.63, GMR_chicken_: 1.36; 95%CI: 0.99-1.85). The association remained when the analysis was restricted to area 4 (GMR_beef_: 1.67; 95%CI: 1.09-2.57, GMR_chicken_: 1.63; 95%CI: 1.04-2.55), but was not present for the areas 1–3. The adjusted R-square from the overall multivariate analysis was 0.43.

**Table 4:** **Association of characteristics of the population with** β**-HCH level (ng/g lipid): GMR from multiple linear regression in the whole population and stratified by sub-area**

	**β-HCH**		**β-HCH**		**β-HCH**	
	**(Area 1–4)**		**(Area 4)**		**(Area 1–3)**	
	**GMR**	**95%CI**	**GMR**	**95%CI**	**GMR**	**95%CI**
**Sex**						
Males	1.00		1.00		1.00	
Females	1.20	0.98-1.47	1.17	0.84-1.65	**1.30**	1.03-1.63
**Age group (years)**						
25-34	1.00		1.00		1.00	
35-44	1.34	0.99-1.80	1.28	0.77-2.13	**1.56**	1.12-2.17
45-54	**1.92**	**1.40-2.62**	1.56	0.94-2.58	**2.48**	1.75-3.53
55-64	**2.76**	**2.04-3.74**	**2.51**	**1.52-4.13**	**3.51**	**2.49-4.95**
**Level of Education**						
Primary school	1.00		1.00		1.00	
Secondary school	**0.78**	**0.62-0.98**	**0.81**	**0.54-**1.21	0.81	0.64-1.02
University degree	0.86	0.57-1.29	0.91	0.41-1.99	0.93	0.63-1.37
Area of residence*						
Area 1	1.00					
Area 2	0.98	0.68-1.41				
Area 3	1.32	0.91-1.93				
Area 4	**2.00**	**1.36-2.94**				
**Any use of well water**						
No	1.00		1.00		1.00	
Yes	**1.42**	**1.08-1.88**	1.46	0.97-2.20	1.42	0.97-2.08
**Provenience of food§**						
Commercial	1.00		1.00		1.00	
Local	1.16	0.89-1.52	1.75	0.97-3.18	1.00	0.78-1.29
Own production	***1.26***	***0.94-1.69***	**2.27**	**1.24-4.13**	0.75	0.55-1.01
**Consume of Beef§**						
None/commercial	1.00		1.00		1.00	
Local	0.91	0.61-1.35	0.84	0.50-1.43	0.81	0.36-1.83
Own production	**1.88**	**1.35-2.63**	**1.67**	**1.09-2.57**	dropped	
**Consume of Chickens§**						
None/commercial	1.00		1.00		1.00	
Local	1.08	0.72-1.60	1.06	0.56**-**1.98	1.33	0.73-2.43
Own production	1.36	0.99-1.85	**1.63**	**1.04-2.55**	0.67	0.36-1.83

*Area 1: reference; Area 2: Colleferro urban/rural; Area 3: industrial; Area 4: river.

GMR: Geometric Mean Ratio from multivariate linear regression model.

95%CI: 95% Confidence Intervals.

§ the model with the variables consume of beef and consume of chicken is alternative to the model with the variable provenience of food.

## Discussion

We found high levels of ß-HCH in a sample of the population living in the Sacco Valley who was examined after the accidental finding of contaminated cow’s milk from the area. The overall geometric mean (GM) was 60.58 ng/g lipid, but the highest levels (GM= 97.65 ng/g lipid) were found in subjects living in the area close to the river (area 4). Age had a strong effect, as older people had higher values, suggesting that the contamination must have begun several decades ago, leading to chronic accumulation in the body.

Dividing the valley into 4 sub-areas (Figure 1) reflected the expected gradient of exposure, and started from the most to the least exposed sub-area. We anticipated that the sub-area within a 1 km linear buffer along the river (area 4) would be the most exposed because of the results of soil and river water tests (Technical report on the Monitoring of environmental matrix and health effects of the population residing in the Sacco Valley. DGR N.540, 19.05.2005 and N.550, 27.05.2005.). Then, we had the 1 km radial buffer around the chemical plant in Colleferro (area 3), where the toxic landfills were found. We used as reference the built-up area of two small towns near Colleferro, Segni and Gavignano (area 1), which are located on a hill (about 500 m altitude) and relatively far from the source of contamination. In fact, we found only one person from these towns with a concentration of ß-HCH higher than the mean value, who reported habitual consumption of cheese produced in a farm along the river. All remaining people were assigned to area 2 that comprised the territory not included in the previous sub-areas, that is the city of Colleferro beyond the 1 km radial buffer around the plant, the rest of Colleferro, Segni and Gavignano more than 1 km from the river and at the bottom of area 1.

We excluded from the study people with characteristics that could have affected their serum levels of ß–HCH, that is people who had resided in the Sacco valley for less than 15 years, to allow for a minimum period of bioaccumulation. For the same reason we also included only people over 24 years of age. We excluded people whose characteristics may have influenced the excretion of the pollutant, relative to its high lipophilic characteristics, such as significant weight loss in a recent period or breastfeeding in the previous 15 years [1]. Finally, people with cancer were exclude for ethical reasons.

As we did not find other studies on populations with long term exposure to ß–HCH from a point-source, we could only compare our data with the results from other surveys on the general population. In 2008 the National Health Institute conducted a biomonitoring study on the serum levels of ß –HCH in people from the general population from three cities, Brescia (northern Italy) Rome (central Italy) and Naples (southern Italy) [19]. The overall mean serum levels of ß –HCH was 29 ng/g lipid, ranging from 18.6 ng/g lipid in the group from central Italy to 41.5 in the group from the northern part of the country. These results are much lower than those found in our study (mean: 99 ng/g lipid, SD: 121.3), suggesting a point source contamination of our study population. In a study of elderly Swedish women (aged 50–74 years) [20], the mean ß-HCH level was 51 ng/g lipid compared to 61 ng/g lipid in the women of our sample. A study conducted in Poland in 2004 found very low levels of ß-HCH (mean: 3.9 ng/g lipid) in women who had just given birth. In a study conducted on a representative sample of the population of Catalonia [17], the geometric mean (in ng/g lipid) of ß-HCH was higher than that found in our population (GM: 83 ng/g lipid) indicating some contamination in that area.

The main determinants of ß-HCH blood levels in our study were age, use of private wells and consumption of local foods. It seems well established that blood concentrations of the pollutant investigated in this study increase with age, and it is assumed that this is the consequence of cumulative exposure and bioaccumulation [8,21,22]. Our results suggest that ß–HCH residuals from the industrial plant landfills contaminated the Sacco river and private well water. Contamination could have occurred directly through private well water, or indirectly through the consumption of local food (mainly meat) made from animals (cows) bred in the contaminated area, which grazed in pastures that had been contaminated by the frequent flooding of the river.

Other studies on different types of pollutants have found local water and food sources to be the primary vehicles of contamination. The C8 Health Project [23,24], conducted in 2005–2006 on a population of 69,000 Ohio and West Virginia residents who lived in six contaminated districts surrounding a chemical plant that released large quantities of perfluorooctanoic acid (PFOA), found that the residential water source was the primary determinant of high serum PFOA concentrations, a result confirmed in the study of the determinants of the C8 Health project [25].

To our knowledge, this is the first example of study on long term non-occupational exposure to a specific organic pollutant such as ß–HCH. We assessed the serum concentration of other organic pollutants, however we did not report the results because they did not reflect specific human contamination as described in the introduction.

Although we identified several potential determinants of ß–HCH blood concentrations, the overall adjusted R-square from our model was not optimal (0.43) indicating that other factors that were not covered in our survey may have been influential. Certainly, individual detoxification abilities together with family characteristics and behaviors were not investigated (only five people had a family relation in this study), but they could have played an important role.

A limit of the study is the lack of information on the Body Mass Index (BMI), that is known to be associated with ß-HCH levels, due to its lipophilic characteristics, and on occupational history. As most of the people residing close to the river should presumably be farmers using, or having used in the past, lindane for their crops, a part of the residual variability in high ß-HCH blood levels could also been explained by the occupational use of this pesticide. In any case, information on BMI and occupational history will be assessed by an on-going epidemiological and health surveillance program in the area, focused on the population living close to the river. In this phase we will also assess more detailed information on food frequencies and source of different kind of foods.

Another limit of the study is that it is based only on a sample of the population residing in the area. Although the sample was randomly selected based on the population register of the three municipalities involved, the blood samples were taken from the first 246 people who were willing to provide them.

The results of our study led the local authorities to establish a long-term epidemiological and health surveillance programme of everyone who resides in the area close to the river (about 600 people), to better understand the modality of exposure and contamination and to assess and follow the physical burden and health status of the population with regard to ß–HCH and its potential effects on health.

## Conclusions

The results of this study show that people living along the Sacco river have been contaminated with ß–HCH, probably through the use of private well water and the consumption of local and privately produced foods. We also think that other factors that have not yet been investigated could help to explain more deeply the modality of contamination. A more accurate analysis of the population in this sub-area, based on the initial results of the ongoing surveillance programme, is under way to better clarify the contamination and its health effects.

## Abbreviations

HCH: Hexachlorocyclohexane; ß-HCH: Beta-hexachlorocyclohexane; γ-HCH: γ-hexachlorocyclohexane; GM: Geometric Mean; GSD: Geometric Standard Deviation; GMR: Geomteric Mean Ratio; 95%CI: 95% Confidence Intervals; ng/g lipid: nanograns/grams of lipid; ss: sandy soil; mL: milliliters; EPA: Environmental Protection Agency; PFOA: Perfluorooctanoic acid.

## Competing interests

The authors declare that they have no competing interests.

## Authors’ contributions

DP participated in the study design, carried out the primary data analysis, and prepared the first draft of the manuscript. FFa participated in the study design, in the coordination and the execution of data collection and in writing the manuscript. EDF participated in the study design and in the manuscript preparation and coordinated the exposure assessment (laboratory analysis). FB participated in the study design, in the coordination of data collection and in writing the manuscript. AA participated in the exposure assessment and in the manuscript preparation. VDO participated in the study design, data management and analysis and manuscript preparation. VF participated in the study design, in the selection of the population sample and in the manuscript preparation. AMI participated in the exposure assessment and in the manuscript preparation. SN participated in the statistical analysis and in the manuscript preparation. FFo participated in the study design, analysis of data and in writing the manuscript. All authors read and approved the final manuscript.

## Supplementary Material

Additional file 1 Descriptive data (Mean, Standard Deviation and Geometric Mean of other pollutants assessed in the biomonitoring survey. Click here for file

Additional file 2 Distribution of the sources for each food by sub-area of residence. Click here for file

Additional file 3 Descriptive data (GM and GSD) of ß -HCH (ng/g lipid) by each kind of food’s source. Click here for file

Additional file 4 Association of food sources for each kind of food with β-HCH level (ng/g lipid): GMR from multiple linear regression in the whole population and in the area close to the river. Click here for file
