# Supplementary material for: A biomonitoring study on blood levels of beta-hexachlorocyclohexane among people living close to an industrial area
Source: Environ Health. 2013 Jul 16;12:57. doi: 10.1186/1476-069X-12-57 (PMC3729409; doi:10.1186/1476-069X-12-57)
Supplement: Additional file 1 — Descriptive data (Mean, Standard Deviation and Geometric Mean of other pollutants assessed in the biomonitoring survey. [file 1476-069X-12-57-S1.doc]

**Descriptive data (Mean, Standard Deviation and Geometric Mean of other pollutants assessed in the biomonitoring survey**

|  |  |  |  |  |  |
| --- | --- | --- | --- | --- | --- |
| **Pollutant** | **Total** | **Area 1** | **Area 2** | **Area 3** | **Area 4** |
|  |  |  |  |  |  |
|  |  |  |  |  |  |
| **α-HCH (ng/g lipid)** |  |  |  |  |  |
| N | 197 | 24 | 45 | 33 | 95 |
| Mean (SD) | 12.38 (12.69) | 9.49 (5.55)* | 9.41 (6.03)* | 9.52 (4.06)* | 15.52 (16.92)* |
| GM | 9.51 | 8.52 | 8.19 | 7.71 | 10.82 |
| **γ-HCH (ng/g lipid)** |  |  |  |  |  |
| N | 197 | 24 | 45 | 33 | 95 |
| Mean (SD) | 14.96 (1.06) | 11.99 (6.21) | 11.59 (6.50) | 13.44 (6.05) | 17.82 (20.01) |
| GM | 11.66 | 10.87 | 10.24 | 11.94 | 12.52 |
| **HCB (ng/g lipid)** |  |  |  |  |  |
| N | 217 | 25 | 46 | 39 | 107 |
| Mean (SD) | 53.17 (4.63) | 44.82 (51.13) | 42.45 (23.36) | 75.62 (132.9) | 51.55 (45.64) |
| GM | 39.02 | 32.96 | 36.21 | 46.02 | 39.47 |
| ***p,p’*-DDE (ng/g lipid)** |  |  |  |  |  |
| N | 215 | 25 | 46 | 38 | 106 |
| Mean (SD) | 459.1 (441.4) | 374.2 (422.9) | 467.6 (399.5) | 546.2 (200.4) | 444.1 (441.4) |
| GM | 304.3 | 249.4 | 335.9 | 357.9 | 288.3 |
| ***p,p’*-DDT (ng/g lipid)** |  |  |  |  |  |
| N | 173 | 25 | 37 | 30 | 81 |
| Mean (SD) | 11.45 (11.20) | 9.33 (5.69) | 9.86 (9.20) | 12.89 (12.32) | 12.30 (12.76) |
| GM | 8.43 | 8.11 | 7.62 | 9.65 | 8.52 |
| **NDL-PCB (ng/g lipid)** |  |  |  |  |  |
| N | 222 | 26 | 46 | 40 | 110 |
| Mean (SD) | 221.8 (138.6) | 236.6 (122.7) | 249.9 (176.3) | 220.8 (117.4) | 206.8 (130.8) |
| GM | 187.1 | 206.0 | 205.6 | 194.1 | 173.6 |
| **DL-PCB (ng/g lipid)** |  |  |  |  |  |
| N | 222 | 26 | 46 | 40 | 110 |
| Mean (SD) | 234.8 (147.1) | 247.4 (127.7) | 262.2 (179.8) | 234.3 (128.6) | 220.5 (142.5) |
| GM | 198.4 | 216.0 | 217.4 | 204.4 | 185.1 |
| **Cadmium** |  |  |  |  |  |
| N | 240 | 30 | 52 | 47 | 111 |
| Mean (SD) | 0.58 (1.66) | 1.17 (4.49) | 0.73 (0.81) | 0.60 (0.55) | 0.35 (0.24) |
| GM | 0.34 | 0.34 | 0.46 | 0.40 | 0.28 |
| **Mercury** |  |  |  |  |  |
| N | 240 | 30 | 52 | 47 | 111 |
| Mean (SD) | 3.90 (3.72) | 4.39 (4.29) | 4.08 (3.44) | 4.52 (5.48) | 3.43 (2.59) |
| GM | 2.99 | 3.20 | 3.25 | 3.30 | 2.71 |
| **Piombo** |  |  |  |  |  |
| N | 240 | 30 | 52 | 47 | 111 |
| Mean (SD) | 33.5 (29.3) | 37.1 (24.9) | 30.9 (17.9) | 28.0 (15.4) | 36.0 (37.8) |
| GM | 27.0 | 30.8 | 26.8 | 24.6 | 27.1 |
|  |  |  |  |  |  |

Area 1: reference; Area 2: Colleferro urban/rural; Area 3: industrial; Area 4: river.

α-HCH: α-hexachlorocyclohexane; γ-HCH: γ-exachlorocyclohexane, HCB: hexachloro-benzene; *p,p’*-DDE: p,p’-Dichlorodiphenyldichloroethylene; *p,p’*-DDT: p,p'-Dichlorodiphenyltrichloroethane; NDL-PCB: Non Dioxin-like Polychlorinated Biphenyls; DL-PCB: Dioxin-like Polychlorinated biphenyls; GM: geometric Mean; SD: Standard Deviation.

* significative p-value from F-test
