# Supplementary material for: A biomonitoring study on blood levels of beta-hexachlorocyclohexane among people living close to an industrial area
Source: Environ Health. 2013 Jul 16;12:57. doi: 10.1186/1476-069X-12-57 (PMC3729409; doi:10.1186/1476-069X-12-57)
Supplement: Additional file 2 — Distribution of the sources for each food by sub-area of residence. [file 1476-069X-12-57-S2.doc]

**Distribution of the sources for each food by sub-**area of residence

| **Variables** | **Area 1** | **Area 2** | **Area 3** | **Area 4** | **Total** |
| --- | --- | --- | --- | --- | --- |
|  | **N (%)** | **N (%)** | **N (%)** | **N (%)** | **N (%)** |
|  |  |  |  |  |  |
| **TOTAL** | **30 (100)** | **52 (100)** | **47 (100)** | **117 (100)** | **246 (100)** |
| **Eggs** |  |  |  |  |  |
| None/commercial | 16 (53.3) | 33 (63.5) | 37 (78.7) | 32 (27.3) | 118 (48.0) |
| Local | 7 (23.3) | 11 (21.1) | 10 (21.3) | 12 (10.3) | 40 (16.2) |
| Own production | 7 (23.3) | 8 (15.4) | 0 (0.00) | 73 (62.4) | 88 (35.8) |
| **Milk** |  |  |  |  |  |
| None/commercial | 30 (100) | 52 (100) | 47 (100) | 100 (85.5) | 229 (93.1) |
| Local | 0 (0.00) | 0 (0.00) | 0 (0.00) | 2 (1.71) | 2 (1.71) |
| Own production | 0 (0.00) | 0 (0.00) | 0 (0.00) | 15 (12.8) | 15 (12.8) |
| **Cheese** |  |  |  |  |  |
| None/commercial | 25 (83.3) | 50 (96.1) | 45 (95.7) | 85 (72.6) | 205 (83.3) |
| Local | 5 (16.7) | 2 (3.9) | 2 (4.3) | 12 (10.3) | 21 (8.54) |
| Own production | 0 (0.00) | 0 (0.00) | 0 (0.00) | 20 (17.1) | 20 (8.13) |
| **Chicken** |  |  |  |  |  |
| None/commercial | 28 (93.3) | 40 (76.9) | 43 (91.5) | 38 (32.5) | 149 (60.6) |
| Local | 0 (0.00) | 6 (11.5) | 4 (8.51) | 11 (9.40) | 21 (8.54) |
| Own production | 2 (6.7) | 6 (11.5) | 0 (0.00) | 68 (58.1) | 76 (30.9) |
| **Beef** |  |  |  |  |  |
| None/commercial | 30 (100) | 51 (98.1) | 44 (93.6) | 56 (47.9) | 181 (73.6) |
| Local | 0 (0.00) | 1 (1.92) | 3 (6.4) | 16 (13.7) | 20 (8.13) |
| Own production | 0 (0.00) | 0 (0.00) | 0 (0.00) | 45 (38.5) | 45 (18.3) |
| **Pork** |  |  |  |  |  |
| None/commercial | 27 (90.0) | 49 (94.2) | 46 (97.9) | 62 (53.0) | 184 (74.8) |
| Local | 1 (3.33) | 1 (1.92) | 1 (2.13) | 11 (9.40) | 14 (5.69) |
| Own production | 2 (6.67) | 2 (3.85) | 0 (0.00) | 44 (37.6) | 48 (19.5) |
| **Liver** |  |  |  |  |  |
| None/commercial | 30 (100) | 51 (98.1) | 47 (100) | 103 (88.0) | 231 (93.9) |
| Local | 0 (0.00) | 0 (0.00) | 0 (0.00) | 4 (3.42) | 4 (1.63) |
| Own production | 0 (0.00) | 1 (1.92) | 0 (0.00) | 10 (8.55) | 11 (4.47) |
| **Raw vegetables** |  |  |  |  |  |
| None/commercial | 19 (43.3) | 26 (50.0) | 37 (78.7) | 29 (24.8) | 111 (45.1) |
| Local | 3 (10.0) | 13 (25.0) | 9 (19.15) | 8 (6.84) | 33 (13.4) |
| Own production | 8 (26.7) | 13 (25.0) | 1 (2.13) | 80 (68.4) | 102 (41.5) |
| **Cooked vegetables** |  |  |  |  |  |
| None/commercial | 21 (70.0) | 30 (57.7) | 33 (70.2) | 27 (23.1) | 111 (45.1) |
| Local | 2 (6.67) | 12 (23.1) | 12 (25.5) | 9 (7.69) | 35 (14.2) |
| Own production | 7 (23.3) | 10 (19.2) | 2 (4.26) | 81 (69.2) | 100 (46.7) |
|  |  |  |  |  |  |
| Area 1: reference; Area 2: Colleferro urban/rural; Area 3: industrial; Area 4: river | | | | | |
