# Supplementary material for: A biomonitoring study on blood levels of beta-hexachlorocyclohexane among people living close to an industrial area
Source: Environ Health. 2013 Jul 16;12:57. doi: 10.1186/1476-069X-12-57 (PMC3729409; doi:10.1186/1476-069X-12-57)
Supplement: Additional file 3 — Descriptive data (GM and GSD) of ß -HCH (ng/g lipid) by each kind of food’s source. [file 1476-069X-12-57-S3.doc]

**Descriptive data (GM and GSD) of ß -HCH (ng/g lipid) by each kind of food’s source**

|  | **N** | **%** | **GM** | **±** | **GSD** | **pvalue*** |
| --- | --- | --- | --- | --- | --- | --- |
| **TOTAL** | 216 |  | 60.58 | ± | 1.00 |  |
| **Eggs** |  |  |  |  |  | <0.0001 |
| None/commercial | 101 | 46.8 | 45.97 | ± | 2.27 |  |
| Local | 36 | 16.7 | 44.09 | ± | 2.22 |  |
| Own production | 79 | 36.6 | 99.64 | ± | 2.83 |  |
| **Milk** |  |  |  |  |  | <0.0001 |
| None/commercial | 199 | 92.1 | 56.24 | ± | 2.55 |  |
| Local | 2 | 0.9 | 27.69 | ± | 2.03 |  |
| Own production | 15 | 6.9 | 180.3 | ± | 2.33 |  |
| **Cheese** |  |  |  |  |  | 0.0004 |
| None/commercial | 177 | 81.9 | 88.06 | ± | 2.59 |  |
| Local | 19 | 8.8 | 58.54 | ± | 2.46 |  |
| Own production | 20 | 9.3 | 136.7 | ± | 2.54 |  |
| **Chicken** |  |  |  |  |  | <0.0001 |
| None/commercial | 128 | 59.3 | 45.29 | ± | 2.30 |  |
| Local | 19 | 8.8 | 48.41 | ± | 1.96 |  |
| Own production | 69 | 31.9 | 110.5 | ± | 2.79 |  |
| **Beef** |  |  |  |  |  | <0.0001 |
| None/commercial | 154 | 71.3 | 47.66 | ± | 2.40 |  |
| Local | 20 | 9.3 | 59.87 | ± | 2.55 |  |
| Own production | 42 | 19.4 | 146.8 | ± | 2.32 |  |
| **Pork** |  |  |  |  |  | <0.0001 |
| None/commercial | 160 | 74.1 | 50.32 | ± | 2.37 |  |
| Local | 13 | 6.0 | 81.32 | ± | 2.87 |  |
| Own production | 43 | 19.9 | 110.6 | ± | 3.00 |  |
| **Liver** |  |  |  |  |  | 0.0191 |
| None/commercial | 202 | 93.5 | 57.99 | ± | 2.60 |  |
| Local | 4 | 1.9 | 67.99 | ± | 3.14 |  |
| Own production | 10 | 4.6 | 140.0 | ± | 2.80 |  |
| **Raw vegetables** |  |  |  |  |  | <0.0001 |
| None/commercial | 99 | 45.8 | 47.89 | ± | 2.36 |  |
| Local | 28 | 13.0 | 43.94 | ± | 1.93 |  |
| Own production | 89 | 41.2 | 87.06 | ± | 2.91 |  |
| **Cooked vegetables** | |  |  |  |  | <0.0001 |
| None/commercial | 98 | 45.4 | 46.99 | ± | 2.34 |  |
| Local | 31 | 14.4 | 44.68 | ± | 1.93 |  |
| Own production | 87 | 40.3 | 89.89 | ± | 2.91 |  |
|  |  |  |  |  |  |  |
| GM: Geometric Mean; GSD: Geometric Standard Deviation; | | | | | | |
| * p-value from F-test performed on the log distribution of ß-HCH | | | | | | |
