# Supplementary material for: A biomonitoring study on blood levels of beta-hexachlorocyclohexane among people living close to an industrial area
Source: Environ Health. 2013 Jul 16;12:57. doi: 10.1186/1476-069X-12-57 (PMC3729409; doi:10.1186/1476-069X-12-57)
Supplement: Additional file 4 — Association of food sources for each kind of food with β-HCH level (ng/g lipid): GMR from multiple linear regression in the whole population and in the area close to the river. [file 1476-069X-12-57-S4.doc]

**Association of food sources for each kind of food with β-HCH level (ng/g lipid): GMR from multiple linear regression in the whole population and in the are close to the river**

|  | **β−HCH (Area 1-4)** | | | |  | **β−HCH (Area 4)** | | | |
| --- | --- | --- | --- | --- | --- | --- | --- | --- | --- |
|  | **GMR*** | **95%CI** | | |  | **GMR*** | **95%CI** | | |
| **Eggs** |  |  |  |  |  |  |  |  |  |
| None/commercial | 1.00 |  |  |  |  | 1.00 |  |  |  |
| Local | 1.01 | 0.76 | - | 1.34 |  | 0.97 | 0.52 | - | 1.82 |
| Own production | **1.46** | **1.12** | **-** | **1.90** |  | **1.75** | **1.16** | **-** | **2.65** |
| **Milk** |  |  |  |  |  |  |  |  |  |
| None/commercial | 1.00 |  |  |  |  | 1.00 |  |  |  |
| Local | 0.44 | 0.16 | - | 1.21 |  | 0.43 | 0.12 | - | 3.15 |
| Own production | **1.94** | **1.31** | **-** | **2.88** |  | **1.95** | **1.21** | **-** | **3.15** |
| **Cheese** |  |  |  |  |  |  |  |  |  |
| None/commercial | 1.00 |  |  |  |  | 1.00 |  |  |  |
| Local | 0.81 | 0.57 | - | 1.16 |  | 0.73 | 0.42 | - | 1.26 |
| Own production | **1.55** | **1.07** | **-** | **2.23** |  | **1.49** | **0.94** | **-** | **2.36** |
| **Chicken** |  |  |  |  |  |  |  |  |  |
| None/commercial | 1.00 |  |  |  |  | 1.00 |  |  |  |
| Local | 0.98 | 0.69 | - | 1.40 |  | 0.92 | 0.51 | - | 2.99 |
| Own production | **1.76** | **1.34** | **-** | **2.31** |  | **2.05** | **1.41** | **-** | **2.99** |
| **Beef** |  |  |  |  |  |  |  |  |  |
| None/commercial | 1.00 |  |  |  |  | 1.00 |  |  |  |
| Local | 1.00 | 0.71 | - | 1.41 |  | 0.99 | 0.62 | - | 1.59 |
| Own production | **2.25** | **1.69** | **-** | **2.99** |  | **2.25** | **1.58** | **-** | **3.20** |
| **Pork** |  |  |  |  |  |  |  |  |  |
| None/commercial | 1.00 |  |  |  |  | 1.00 |  |  |  |
| Local | 0.97 | 0.63 | - | 1.48 |  | 1.00 | 1.15 | - | 2.40 |
| Own production | **1.55** | **1.17** | **-** | **2.07** |  | **1.66** | **1.15** | **-** | **2.40** |
| **Liver** |  |  |  |  |  |  |  |  |  |
| None/commercial | 1.00 |  |  |  |  | 1.00 |  |  |  |
| Local | 0.65 | 0.31 | - | 1.37 |  | 0.66 | 0.26 | - | 1.66 |
| Own production | 1.54 | 0.95 | - | 2.50 |  | 1.56 | 0.85 | - | 2.85 |
| **Raw vegetables** |  |  |  |  |  |  |  |  |  |
| None/commercial | 1.00 |  |  |  |  | 1.00 |  |  |  |
| Local | 0.89 | 0.64 | - | 1.22 |  | 0.58 | 0.28 | - | 1.19 |
| Own production | 1.09 | 0.84 | - | 1.40 |  | 1.31 | 0.87 | - | 1.97 |
| **Cooked vegetables** |  |  |  |  |  |  |  |  |  |
| None/commercial | 1.00 |  |  |  |  | 1.00 |  |  |  |
| Local | 0.84 | 0.62 | - | 1.43 |  | 0.59 | 0.29 | - | 1.19 |
| Own production | 1.10 | 0.84 | - | 33.4 |  | 1.34 | 0.32 | - | 2.04 |
|  |  |  |  |  |  |  |  |  |  |
| * GMR was adjusted by gender, age class, area of residence, any use | | | | | | | | | |
| of well waters, and level of education | | | | |  |  |  |  |  |
